# Immunologic Interplay Between HIV/AIDS and COVID-19: Adding Fuel to the Flames?

**DOI:** 10.1007/s11904-023-00647-z

**Published:** 2023-01-21

**Authors:** Matteo Augello, Valeria Bono, Roberta Rovito, Camilla Tincati, Giulia Marchetti

**Affiliations:** Clinic of Infectious Diseases and Tropical Medicine, Department of Health Sciences, San Paolo Hospital, ASST Santi Paolo E Carlo, University of Milan, Via A. Di Rudinì, 8, 20142 Milan, Italy

**Keywords:** HIV, AIDS, SARS-CoV-2, COVID-19, Vaccines, Immune responses

## Abstract

**Purpose of Review:**

HIV/AIDS and COVID-19 have been the major pandemics overwhelming our times. Given the enduring immune disfunction featuring people living with HIV (PLWH) despite combination antiretroviral therapy (cART), concerns for higher incidence and severity of SARS-CoV-2 infection as well as for suboptimal responses to the newly developed vaccines in this population arose early during the pandemics. Herein, we discuss the complex interplay between HIV and SARS-CoV-2, with a special focus on the immune responses to SARS-CoV-2 natural infection and vaccination in PLWH.

**Recent Findings:**

Overall, current literature shows that COVID-19 severity and outcomes may be worse and immune responses to infection or vaccination lower in PLWH with poor CD4 + T-cell counts and/or uncontrolled HIV viremia. Data regarding the risk of post-acute sequelae of SARS-CoV-2 infection (PASC) among PLWH are extremely scarce, yet they seem to suggest a higher incidence of such condition.

**Summary:**

Scarce immunovirological control appears to be the major driver of weak immune responses to SARS-CoV-2 infection/vaccination and worse COVID-19 outcomes in PLWH. Therefore, such individuals should be prioritized for vaccination and should receive additional vaccine doses. Furthermore, given the potentially higher risk of developing long-term sequelae, PLWH who experienced COVID-19 should be ensured a more careful and prolonged follow-up.

## Introduction: HIV/AIDS and COVID-19, a Tale of Two Intersecting Pandemics

HIV/AIDS and COVID-19 have been the major pandemics overwhelming the contemporary era. HIV/AIDS was firstly reported in 1981 in previously healthy young men who developed a life-threatening *Pneumocystis carinii* (now known as *Pneumocystis jirovecii*) pneumonia, whose underlying cause was revealed 2 years later to be a retrovirus, which was named human immunodeficiency virus (HIV). Its incidence has been declining since 1996, when protease inhibitors were rolled out for HIV treatment, so that the pandemic may be now considered to have reached an endemic state worldwide [1]. COVID-19 emerged in late 2019 as a severe respiratory disease caused by the quickly identified severe acute respiratory coronavirus 2 (SARS-CoV-2), intersecting the HIV/AIDS pandemics in various ways.

Physicians and medical infrastructures previously dedicated to the care of people living with HIV (PLWH) were reallocated on the frontline in tackling COVID-19 [2]. In-person clinical visits were discouraged in favor of telemedicine, which is most likely destined to become the standard of care for PLWH with well-controlled infection in the future [3]. Limitations also involved HIV infection laboratory monitoring, as laboratory facilities and personnel were employed in diagnostic testing for SARS-CoV-2. Shortage of medical resources, fear of exposure to SARS-CoV-2, and lockdown restrictions hindered the access to antiretroviral therapy [4]. Overall, notwithstanding different approaches to cope with COVID-19 in PLWH have been put forward in different settings, all the aforementioned elements resulted in a suboptimal care of HIV infection and other comorbidities in PLWH and exacerbated inequalities for key populations affected by HIV [5].

In the meanwhile, given the HIV-driven immune dysfunction also in the course of effective combination antiretroviral therapy (cART), concerns for higher susceptibility of PLWH to SARS-CoV-2 infection and poor COVID-19 outcomes arose, thus leading to prioritize this population for vaccine administration when they became available, albeit the readiness of their immune system to respond to these novel vaccines was unknown at the time. After nearly 3 years into the COVID-19 pandemics, numerous studies aiming to unveil whether PLWH are actually at higher clinical risk have been published, but yielded apparently contradictory findings. Additionally, data assessing how efficiently the immune system of PLWH faces SARS-CoV-2 infection and responds to COVID-19 vaccines remain limited.

In this review, we summarize the knowns and the unknowns of the complex interplay between HIV and SARS-CoV-2 infection, with a special focus on the immune responses to SARS-CoV-2 natural infection and vaccination in PLWH.

## Incidence and Clinical Outcome of SARS-CoV-2 Infection in PLWH

An early literature review reported that the prevalence of SARS-CoV-2 infection in PLWH was similar to that observed in the general population [6]. The same study also reported that PLWH accounted for approximately 1.0% of total hospitalized COVID-19 cases [6]. A recent cross-sectional study on 4400 consecutive PLWH attending an HIV Clinic in Spain between November 2020 and May 2021 demonstrated that the prevalence of SARS-CoV-2 infection, determined through antibody presence, was 28% [7]. In contrast, recent findings from the EuroSIDA cohort reported positive SARS-CoV-2 PCR results in 122/1026 participants (1.8%) [8]. The different testing procedures and reasons for testing may explain, at least in part, the opposing results.

From a pathogenic standpoint, single-cell transcriptomic analysis across different tissues reported higher co-expression of *ACE2* and *TMPRSS2* in the lung of HIV-infected humans and in the gut of SHIV-infected non-human primates as compared with uninfected controls [9], suggesting a possible higher risk of SARS-CoV-2 acquisition in PLWH. However, current literature [6, 10–13] indicates that HIV infection per se is not a risk factor for SARS-CoV-2 infection.

Conflicting data have also emerged in terms of clinical outcome of COVID-19 in the setting of HIV infection. One of the first studies published during the first pandemic wave demonstrated that PLWH were at increased risk of mortality among subjects hospitalized for COVID-19 in the UK [14]. An even higher risk of mortality in PLWH was reported when analyzing data from a large primary care database in the same country [15]. A study from Chile, which was also conducted in the first wave, showed that PLWH were more likely to be admitted to the ICU [16]. In keeping with these results, current findings from the WHO Global Clinical COVID-19 platform, which however mirror data contribution from Africa, showed that HIV infection was an independent risk factor for severe COVID-19 and in-hospital mortality [17]. In this respect, some studies [18]*,* but not others [16], reported poor viro-immunological control as a reason underlying disease severity/mortality in PLWH.

In contrast to the above, research has also demonstrated a similar outcome in PLWH and the general population [19–21], highlighting the role of comorbidities in the development of critical COVID-19 [22]. In particular, one study demonstrated a lower rate of ICU admissions, invasive mechanical ventilation, or death in a large cohort of PLWH who were younger than uninfected controls [23]. A recent meta-analysis reported no difference in the risk of death in PLWH compared with the HIV-seronegative population [24]*.*

Taken together, literature published thus far has shown heterogeneity in COVID-19 severity in the context of HIV infection. Age and comorbidities, as well as the lack of viro-immunological control, represent possible risk factors for a worse clinical outcome, and their respective contribution should be assessed in future studies [25•].

Interestingly, some observational studies reported a protective effect of certain antiretroviral drugs, namely tenofovir disoproxil fumarate/emtricitabine (TDF/FTC), against SARS-CoV-2 infection and COVID-19–related outcomes in both PLWH [13, 26, 27] and HIV pre-exposure prophylaxis (PrEP) users [28, 29]. This potential protective effect is biologically plausible due to the ability of nucleotide analog reverse transcriptase inhibitors (NRTIs) to inhibit the SARS-CoV-2 RNA-dependent RNA polymerase [30, 31]. However, others did not find such an association [7, 32]. These contradictory findings may be the consequence of the baseline characteristics of TDF/FTC-users, which are intrinsically associated with a more favorable outcome of SARS-CoV-2 infection.

Data regarding post-acute sequelae of SARS-CoV-2 infection (PASC) in PLWH are limited. The major risk factors for PASC have been described to be severity of disease, being unvaccinated against SARS-CoV-2, and medical comorbidities [33]. Furthermore, PASC has been associated with a residual inflammation following SARS-CoV-2 infection [34]. Therefore, given the potential for greater risk of COVID-19 severity and reduced responses to SARS-CoV-2 vaccines, and considered the higher burden of comorbidities [35] and the greater baseline levels of immune activation and systemic inflammation [36, 37], PLWH may be at higher risk of developing PASC. Actually, the few published studies showed a significantly greater risk of PASC in PLWH when adjusting for other factors, indicating that HIV infection may be an independent risk factor for such condition [38, 39•].

## Immune Responses to SARS-CoV-2 Infection in PLWH

### Innate Immune Responses

Type-I interferons (IFNs-I) constitute the first innate immune barrier to SARS-CoV-2 infection at mucosal sites [40]. However, SARS-CoV-2 has evolved several mechanisms to evade such host defense via both its structural and non-structural proteins [41, 42]. Moreover, inborn mutations in genes involved in the regulation of IFNs-I immunity, as well as production of auto-antibodies against IFNs-I during COVID-19, have been associated with poor clinical outcome [43, 44].

HIV infection triggers interferon responses in the earliest phases, contributing to limit viral replication; nonetheless, persistent exposure to IFNs-I in the chronic phase of HIV infection is associated with desensitization and immune hyperactivation, thus paradoxically contributing to disease progression [45, 46].

**Fig. 1:**
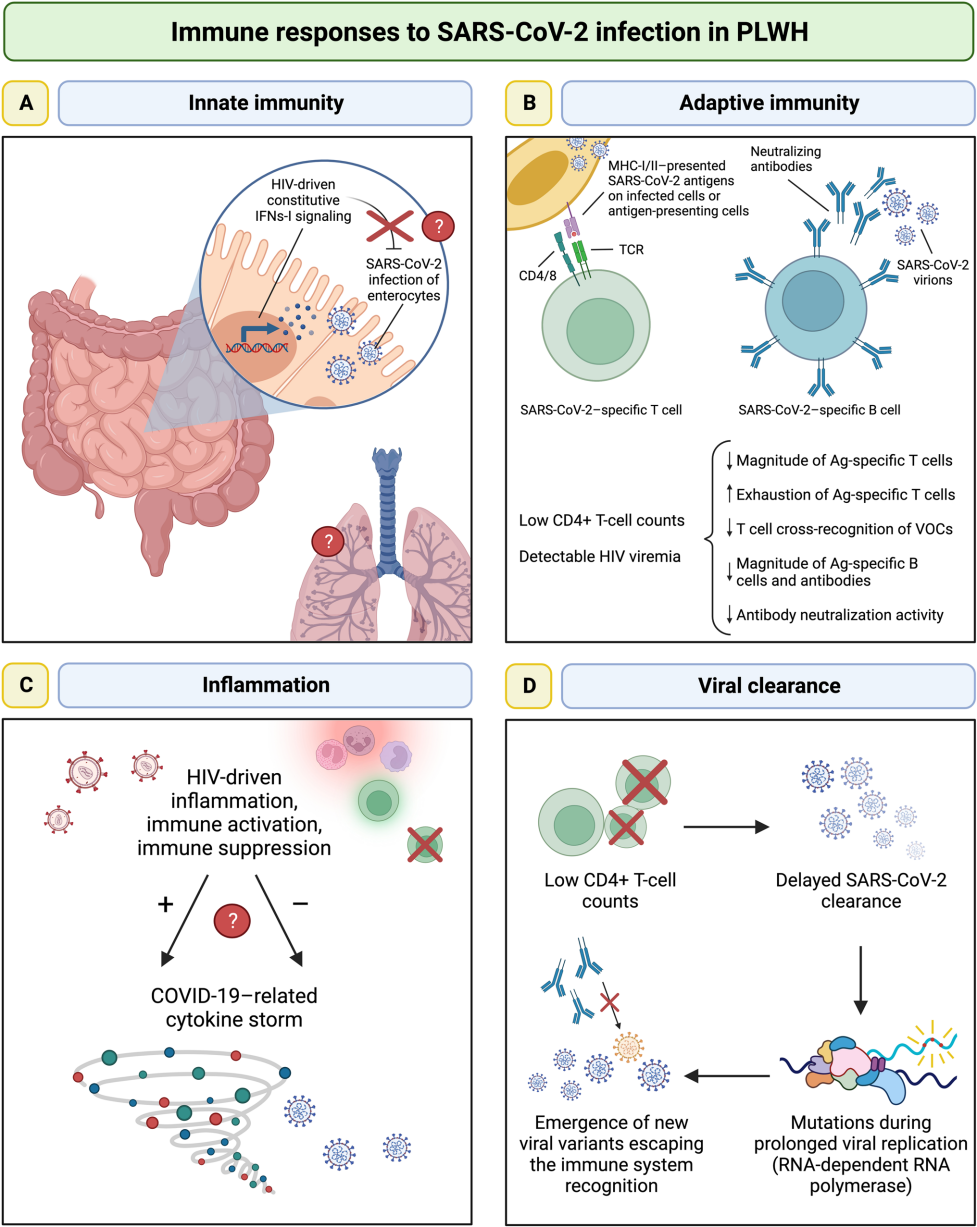
Immune responses to SARS-CoV-2 infection in PLWH. **A** Chronic HIV infection on cART drives a strong constitutive IFN-I signaling within absorptive enterocytes in the small intestine, which, however, is not able to prevent SARS-CoV-2 infection in this compartment. The interplay between HIV and SARS-CoV-2 in modulating IFNs-I responses in other anatomical sites, especially the respiratory tract, which is the site of initial infection, is currently unknown. **B** PLWH with well-controlled HIV infection mount adaptive immune responses comparable to those of people without HIV. Yet, PLWH with low CD4 + T-cell counts and/or detectable HIV viremia develop suboptimal immune responses to SARS-CoV-2 infection (fewer and exhausted SARS-CoV-2–specific T-cells, impaired T-cell cross recognition of VOCs, lower SARS-CoV-2–specific B-cells and neutralizing antibodies). **C** HIV infection is associated with enduring systemic inflammation and immune activation despite effective cART; on the other side, in absence of cART, HIV infection leads to immune suppression, mainly—albeit not only—driven by the depletion of CD4 + T-cells. Whether such features of HIV infection may exacerbate or hinder the COVID-19–related cytokine storm is currently unknown, as the few available data are conflicting. **D** PLWH with low CD4 + T-cell counts may have a delayed SARS-CoV-2 clearance due to the immune defects. The subsequent prolonged viral replication (sustained by the RNA-dependent RNA-polymerase) can lead to the emergence of multiple mutations and thus to the development of new viral variants escaping from antibody neutralization. *PLWH*, people living with HIV; *cART*, combination antiretroviral therapy; *IFN-I*, type-I interferon; *VOCs*, variants of concern. Created with *BioRender.com*

These observations suggest that complex interactions between the two viruses might influence IFNs-I responses in HIV/SARS-CoV-2 coinfection. An interesting study evaluated the impact of HIV infection on gut epithelial cells susceptible to SARS-CoV-2 infection, showing that chronic-treated HIV infection drives a strong interferon signaling response within absorptive enterocytes, which, however, does not prevent SARS-CoV-2 infection in this compartment [47•] (Fig. 1A). These findings may indicate that the persistent IFNs-I signaling which features chronic HIV infection despite effective cART is not able to confer a protection against SARS-CoV-2 infection. However, the interplay between HIV and SARS-CoV-2 in modulating IFNs-I responses in other anatomical sites, especially the upper respiratory tract which is the site of initial infection, and its role in influencing the susceptibility to SARS-CoV-2 infection in PLWH, have not been characterized and require future research efforts.

### Adaptive Immune Responses

Early and robust development of SARS-CoV-2–specific cell-mediated immunity and neutralizing antibodies has been associated to a favorable clinical course of COVID-19 [48–50]. HIV infection is characterized by a profound disruption of the adaptive immune system, in both its cellular and humoral components, with destruction of CD4 + T-cells, increased CD8 + T-cells, T-cell activation/exhaustion, defective T follicular helper (Tfh) cells activity, and dysfunction and polyclonal activation of B-cells [51, 52]. Furthermore, it has recently been demonstrated that SARS-CoV-2 is able to productively infect CD4 + T-cells binding to cell entry receptors other than ACE2 (like LFA-1 and CD147) and to induce their apoptosis which is probably dependent on mitochondria ROS-hypoxia pathways [53••, 54••], suggesting that HIV and SARS-CoV-2 may collide on the immune system. These observations raise the concern that PLWH, especially those with incomplete immune restoration despite virologically effective cART [55], may not appropriately respond to SARS-CoV-2 infection. Thus far, data on adaptive immune responses to natural SARS-CoV-2 infection in this population is limited and, at times, conflicting.

One of the earliest studies in this regard evaluated the T-cell profile in the course of HIV/SARS-CoV-2 coinfection during the first wave of the COVID-19 pandemics, when some PLWH suspended cART due to medication shortages. Compared to SARS-CoV-2 mono-infected individuals, HIV/SARS-CoV-2–co-infected individuals showed reduced Th1 cells and cytotoxic CD8 + T-cell responses, as well as a higher rate of T-cell exhaustion, which was even more pronounced in those not taking cART [56]. These data suggest a synergic effect of HIV and SARS-CoV-2 on T-cell dysfunction, especially in PLWH with uncontrolled HIV viremia, which may not mount proper immune responses against SARS-CoV-2. A more recent study showed that individuals with unsuppressed HIV infection mount weak antigen-specific CD4 + and CD8 + T-cell responses to SARS-CoV-2 and poorly recognize SARS-CoV-2 beta variant, due to HIV-induced immune defects such as low CD4 + T-cell counts, high HIV plasma viral loads, and elevated immune activation. Yet, virologically suppressed PLWH exhibit SARS-CoV-2–specific T-cell responses similar to those of HIV-negative peers, highlighting the role of uncontrolled HIV infection in hampering immune responses to SARS-CoV-2 and T-cell cross-recognition between viral variants, thus partly explaining the high propensity for severe COVID-19 among PLWH and their vulnerability to emerging SARS-CoV-2 VOCs [57••].

Also, inadequate immune reconstitution on virologically effective cART has been shown to potentially hinder the development of T-cell responses to SARS-CoV-2 infection. Indeed, while T-cell responses against structural and non-structural SARS-CoV-2 proteins in the convalescent phase of mild COVID-19 are similar in PLWH with cART-suppressed HIV viral load and HIV-negative subjects overall, the magnitude of SARS-CoV-2–specific T-cell responses is positively related with the CD4/CD8 ratio and the size of naïve CD4 T-cell pool in PLWH [58••]. Accordingly, another study found that HIV infection does not significantly alter the functional and phenotypical profile of SARS-CoV-2–specific CD4 + T-cells, yet the magnitude of SARS-CoV-2–specific T-cell and humoral responses is lower in PLWH with poor CD4 T-cell recovery despite cART [59].

Donadeu et al. reported that COVID-19–recovered PLWH with well-controlled HIV infection are capable of developing a robust adaptive SARS-CoV-2–specific immune response which persists up to 6 months, similar to people without HIV; furthermore, immune responses are more pronounced among severe COVID-19 patients, irrespective of HIV status [60••]. These data suggest that magnitude and persistence of the immune response after SARS-CoV-2 infection may be mainly driven by the degree of COVID-19 clinical severity, rather than the HIV status.

Some studies showed that T-cell and humoral responses to SARS-CoV-2 infection do not necessarily move in the same direction. Peluso et al. found that, in the backdrop of similar humoral responses compared to HIV-uninfected individuals, cART-treated PLWH recovering from SARS-CoV-2 infection display high expression of the co-inhibitory receptor PD-1 on SARS-CoV-2–specific memory CD4 + T-cells and low frequencies of specific CD8 + T-cells, suggesting that they may have impaired T-cell functionality upon reencountering infection [39•]. It has also been reported that although PLWH on effective cART may present lower memory T-cell responses against SARS-CoV-2 as well as dysregulated T follicular helper (Tfh) populations, that is enough to generate a cooperation between T-cells and B-cells that allows to elicit an effective antibody response against the pathogen [61].

Studies evaluating humoral immune responses to SARS-CoV-2 in PLWH also yielded inconsistent observations, probably due to the different demographic and viro-immunological characteristics of study participants, various degree of COVID-19 severity, and sampling during diverse phases post-infection.

Alrubayyi et al. found comparable antibody titers against S1 and N proteins of SARS-CoV-2 in HIV-positive and -negative subjects after mild COVID-19 [58••]. Similarly, Alcaide et al. showed that antibody responses during the 6-month period post-mild COVID-19 do not differ by HIV status [62]. Snyman et al. reported that magnitude, kinetics, and durability of anti-SARS-CoV-2 IgM, IgG, and IgA, as well as neutralization potency, are similar in PLWH and people without HIV [63]. It should be specified that all the studies mentioned above included individuals with well-controlled HIV infection.

In sharp contrast, Spinelli et al. found lower RBD-specific IgG concentrations and pseudovirus neutralizing antibodies titers in PLWH with past SARS-CoV-2 infection as compared to HIV-negative individuals [64]. Liu et al. described that in the acute phase of COVID-19, PLWH exhibit a lower IgG seroconversion rate and shorter duration of humoral responses compared to HIV-negative individuals [65]. It must be noted, however, that both studies included also virally unsuppressed people. Accordingly, Khan et al. showed lower neutralization of the Delta variant and a higher frequency of non-responders in PLWH, with the highest frequency of non-responders in those with uncontrolled HIV viremia; furthermore, neutralization activity was correlated with CD4 + T-cell counts, underscoring the importance of both immune recovery and HIV viremia suppression on cART, in influencing humoral immune responses to SARS-CoV-2 [66].

Humoral responses in the convalescent phase of SARS-CoV-2 infection in PLWH may also be influenced by the severity of COVID-19, since antibodies’ magnitude and functionality in PLWH have been reported similar to those of people without HIV in the mild (asymptomatic) and severe (symptomatic requiring hospitalization) disease, but diminished in the moderate (symptomatic not requiring hospitalization) disease [67].

Lastly, given that PLWH have been shown to be at higher risk of developing PASC, Peluso et al. explored immunologic features potentially related to such condition in this population, yet no relationships between PASC and SARS-CoV-2–specific humoral and T-cell responses or immune exhaustion were found [39•], suggesting that other factors may be involved in PASC pathogenesis in PLWH.

Taken together, these data indicate that adaptive immune responses to SARS-CoV-2 infection in PLWH are similar to those of the general population overall, but may be less efficient in the setting of scarce immune recovery and uncontrolled HIV viremia (Fig. 1B).

### Inflammation

HIV infection elicits a chronic hyperinflammatory state that persists despite effective cART [36, 37], sharing inflammatory markers that have been also described as elevated in severe COVID-19, such as IL-6 and TNF-α [49]. Given these premises, it can be speculated that HIV may exacerbate COVID-19–related cytokine storm and thus severity, leading to unfavorable outcomes. On the other hand, it may be assumed that PLWH can be protected from hyper-activation/inflammation–mediated immunopathology due to the HIV-driven immune defects. As described in HIV-negative individuals [50], PLWH hospitalized for COVID-19 have been shown capable of mounting a profound inflammatory reaction in response to SARS-CoV-2 coinfection that is higher in fatal cases [68]. While some studies reported lower levels of inflammatory markers such as IL-6, TNF-α, and IL-8 in PLWH as compared to the HIV-negative counterpart [61, 69], another study found higher IL-6, TNF-α, and IP-10 in HIV/SARS-CoV-2–coinfected individuals [39•]. Hence, whether immune imbalances which feature chronic HIV infection may enhance or hinder the COVID-19–related cytokine storm is yet to be determined (Fig. 1C).

### Delayed Clearance of SARS-CoV-2 and Immune Escape

Low CD4 + T-cell counts in PLWH can potentially hamper SARS-CoV-2 clearance, as suggested by a murine model of acute SARS-CoV-2 infection, in which depletion of CD4 + T-cells led to reduced antibody response and delayed viral clearance [70]. Prolonged infections may allow SARS-CoV-2 to evolve diverse resistance mutations, since it mutates at a relatively slow rate compared to other RNA viruses due to its proof-reading mechanism [71]. Actually—as previously described in patients with immune suppression due to other causes [72–74]—several cases of prolonged SARS-CoV-2 infection have been reported in PLWH with severe T-cell depletion and/or AIDS, with subsequent emergence of a multitude of mutations conferring extensive escape from antibody neutralization elicited by both ancestral SARS-CoV-2 infection and vaccines [18, 75••, 76, 77] (Fig. 1D).

## SARS-CoV-2 Vaccine Efficacy and Safety in PLWH

When SARS-CoV-2 vaccines were rolled out, PLWH—especially those with current CD4 + T-cell count < 200/μL, evidence of an opportunistic infection, and/or with a detectable viral load—were prioritized for vaccination, due to the potential higher risk for worse COVID-19 outcomes [78].

Given that suboptimal and less durable immune responses to several vaccines have been reported in PLWH, particularly in those with scarce immune recovery despite cART [79–82], concerns were raised about immunogenicity and clinical efficacy of these vaccines in this potentially more vulnerable population.

Unfortunately, the larger phase 2/3 SARS-CoV-2 vaccine clinical trials included few PLWH (176 for mRNA-1273, 196 for BNT162b2, and 107 for ChAdOx1-S), therefore lacking to report efficacy, safety, and immunogenicity for this sub-population [83–85]. Additionally, there are no head-to-head comparisons between different COVID-19 vaccines in PLWH thus far; hence, whether a certain vaccine platform is more effective and should therefore be preferred in this population is currently unknown. Furthermore, data on vaccine-induced protection against emerging variants of concern (VOCs) in this population are also lacking and should be addressed by future research.

Nonetheless, two large longitudinal studies carried out in the USA found a higher rate of breakthrough infections in fully vaccinated PLWH [86, 87]; however, among them, high CD4 + T-cell counts (> 500/μL) and having received an additional dose were associated with a reduced risk [86]. These data clearly suggest that the clinical efficacy of the primary vaccine cycle may be inferior in PLWH, especially in those with low CD4 + T-cell counts, who should therefore receive additional doses.

Risk of severe side effects to SARS-CoV-2 vaccines has not been reported to be higher in PLWH than in general population. However, HIV viral blips after SARS-CoV-2 vaccination were described in some PLWH with low CD4 + T-cell *nadir* and/or high HIV-RNA *zenith* [88, 89]. Previous studies have found transient increases in HIV viral load with other vaccines, including HBV, influenza, and *Streptococcus pneumoniae* [90–92], which typically occurs within 7–14 days post-vaccination [93]. Such phenomenon may be attributed to a reactivation of the latent HIV reservoir, probably due to a vaccine-elicited generalized inflammatory response with cytokine production able to activate bystander cells harboring latent HIV, rather than the solely activation of HIV-infected vaccine-specific T-cells [93]. In this context, the increased HIV transcription is accompanied by enhanced HIV-specific CD8 + T-cell responses, pointing to standard vaccines as a potential tool to reverse HIV latency to enable eradication by cytotoxic T-cells [93]. Accordingly, a recent study showed that BNT162b2 vaccine activates the RIG-I/TLR–TNF–NFkB axis, resulting in transcription of HIV proviruses; in parallel, Nef-specific CD8 + T-cells increase and acquire cytotoxic effector functions, which correlate with reduction of cell-associated HIV-mRNA, suggesting killing or suppression of cells transcribing HIV; however, significant depletion of intact proviruses was not observed, highlighting challenges to achieving HIV reservoir reductions [94•]. Nevertheless, the interplay between vaccines, immune system, and latent HIV infection is yet to be thoroughly understood and deserves further research in order to inform future HIV eradication strategies.

## Immune Responses to SARS-CoV-2 Vaccines in PLWH

Whilst published data on clinical efficacy in PLWH are scarce, immunogenicity to SARS-CoV-2 vaccines have been studied more extensively albeit not comprehensively. In particular, cellular responses to these vaccines have been evaluated only by few studies thus far, likely due to technical difficulties; however, given the T-cell dysfunction which features HIV infection, it would be of paramount importance to assess the ability of COVID-19 vaccines to induce polyfunctional SARS-CoV-2–specific T-cells, which have been shown to lend protection against the severe forms of disease [48, 49].

In general, immune responses to mRNA vaccines (mRNA-1273 and BNT162b2) have been reported similar to those of the general population in people with well-controlled HIV infection [95•, 96••]. Additionally, no differences in BNT162b2-elicited antibody neutralization of different VOCs (alpha, beta, and gamma) have been shown between PLWH and HIV-negative vaccinees [97].

On the contrary, PLWH with low CD4 + T-cell counts, detectable viremia, and/or previous AIDS were found to have weaker and less durable humoral and T-cell responses to mRNA vaccines [95•, 96••, 98•, 99, 100, 101•], suggesting that they may benefit from additional vaccine doses. In this respect, a third dose of a mRNA vaccine following the primary cycle has been shown to strongly boost humoral albeit not T-cell responses in PLWH with advanced disease at the time of HIV diagnosis (CD4 + T-cells < 200/μL and/or AIDS), irrespective of the current CD4 + T-cell count [102••]. Furthermore, PLWH with a current CD4 + T-cell count < 250/μL retain a similar neutralization activity against Delta variant, yet reduced against Beta [103].

In accordance with these findings, we have recently shown that in PLWH with pre-cART advanced immunodeficiency and full virologic control on cART, a 2-dose mRNA-1273 vaccine cycle is able to induce spike-specific memory polyfunctional T helper 1 (Th1) and T follicular helper (Tfh) cells as well as anti-RBD antibodies capable of inhibiting the spike-ACE2 binding. Such vaccine-elicited immune responses, which are still detectable after 6 months from vaccination, are not inferior to those of HIV-negative peers, albeit a positive correlation between humoral responses and CD4 + T-cell recovery on cART were found [104].

As regard adenoviral vector vaccines (ChAdOx1-S and Ad26.COV2.S), no significant differences were found in magnitude and durability of vaccine-induced humoral and T-cell responses based on HIV status [66, 105••, 106]. PLWH also show ChAdO1x-S–elicited cross-reactive binding antibodies to the Beta variant; furthermore, those who develop high-titre responses also retain neutralization activity against Beta [107]. PLWH with well-controlled HIV vaccinated with Ad26.COV2.S display similar neutralization response of the Delta variant as compared to people without HIV [66]. However, immunogenicity of such vaccines in PLWH with lower CD4 + T-cell counts has not been evaluated.

Alternative vaccine platforms—protein (NVX-CoV2373) and inactivated vaccines (CoronaVac, BBIBP-CorV)—have been administered in PLWH in some countries. Humoral responses to NVX-CoV2373 have been shown lower in PLWH as compared to people without HIV, especially in those without prior SARS-CoV-2 infection [108•]. Immunogenicity of inactivated vaccines in PLWH have been reported similar [109, 110•] or lower [111–113] than in general population according to different studies, but invariably reduced in those with low CD4 + T-cell counts and/or CD4/CD8 ratio [109, 110•, 111].

A comparison between the immunogenicity of different vaccine platforms in PLWH has been reported in some studies. mRNA vaccines appear to elicit the highest immune responses in this population [113–115, 116•, 117]. Among mRNA vaccines, BNT162b2 has been show less immunogenic than mRNA-1273 [100, 102••, 118]. However, given the lack of immune thresholds that correlate to protection after vaccination, the clinical significance of such lower immunogenicity is currently unknown. Nonetheless, in light of these data and awaiting future clarifications on their clinical relevance, mRNA vaccines should be preferred in PLWH [119].

Some studies reported higher immune responses to both mRNA and adenoviral vector COVID-19 vaccines in PLWH with previous SARS-CoV-2 infection [107, 118, 120] or preexisting cross-reactive T-cell responses correlated with prior exposure to seasonal coronaviruses [106], suggesting that, similar to what observed in the general population, a preexisting immune memory may boost immune responses to COVID-19 vaccines.

Lastly, similarly to HIV-negative individuals [121, 122], PLWH show waning antibody immunity (especially against VOCs), yet persistent T-cell responses 6 months post-vaccination [123•], pointing to a potential role of vaccine-elicited cellular immune memory in ensuring a long-term protection.

The main findings of the major studies evaluating immune responses to different SARS-CoV-2 vaccine platforms in PLWH are summarized in Table 1.

**Table 1:** Main findings of the major studies evaluating immune responses to different SARS-CoV-2 vaccine platforms in PLWH

Reference	Vaccine (doses)	Study design (country)	Participants characteristics	Immunovirological parameters of PLWH	Prior SARS-CoV-2 infection	Immunological parameter (assay)	Main findings
Nault et al*.* [95•]	PLWH: mRNA-1273 (1 dose) HIV-negative controls: BNT162b2 (1 dose)	Observational study (Canada)	106 PLWH: 90 (84.9%) males, 16 (15.1%) females; median age: 43 years (IQR: 21–65) 20 HIV-negative controls: 7 (35%) males, 13 (65%) females; median age: 47 years (IQR: 21–59)	CD4 + T-cell count strata: - < 250/μL: *n* = 6 - 250–500/μL: *n* = 18 - > 500/μL: *n* = 82 Detectable plasma HIV viral load in 4 PLWH	Individuals with prior SARS-CoV-2 infection excluded	Anti-RBD IgG (ELISA)	In PLWH with CD4 + T-cell counts > 250/μL anti-RBD IgG response comparable to HIV-negative controls Lower humoral responses in PLWH with CD4 + T-cell counts < 250/μL
Lombardi et al*.* [120]	mRNA-1273 (2 doses)	Prospective cohort study (Italy)	71 PLWH: 60 (84.5%) males, 11 (15.5%) females; median age: 47 years 10 HIV-negative controls: 7 (70%) males, 3 (30%) females; median age: 58 years	Median CD4 + T-cell count: 747/μL (IQR: 593–942) CD4 + T-cell count strata: - < 350/μL: *n* = 6 (8.4%) - 350–500/μL: *n* = 7 (9.8%) - > 500/μL: *n* = 58 (81.7%) Plasma HIV-RNA < 50 copies/mL in all participants	Prior SARS-CoV-2 infection (confirmed by anti-N Ig) in 9 PLWH and 2 HIV-negative controls (SARS-CoV-2–experienced)	Anti-RBD total Ig (ELISA) Neutralizing antibody activity (pseudovirus neutralization assay)	Both anti-RBD Ig and neutralizing activity comparable to those of HIV-negative controls, even when stratified according to the CD4 + T-cell count Higher anti-RBD Ig and neutralizing antibody activity in SARS-CoV2–experienced PLWH compared to SARS-CoV-2–naïve
Antinori et al*.* [96••]	PLWH: 93 (57%) BNT162b2, 70 (43%) mRNA-1273 (2 doses) HIV-negative controls: BNT162b2 (2 doses)	Observational study (Italy)	166 PLWH, described according to CD4 + T-cell count strata: > 500/μL: 12.8% females; median age: 54 years (IQR: 46–59) 200–500/μL: 16.1% females; median age: 54 years (IQR: 46–59) < 200/μL: 25/μL females; median age: 57 years (IQR: 52–60) 169 HIV-negative controls: 71.6% females; median age: 42 years (IQR: 32–53)	CD4 + T-cell count strata: - > 500/μL: *n* = 78 - 200–500/μL: *n* = 56 - < 200/μL: *n* = 32 Plasma HIV-RNA < 50 copies/mL: - > 500/μL: *n* = 78 (100%) - 200–500/μL: *n* = 52 (92.9%) - < 200/μL: *n* = 22 (68.8%)	Individuals with prior SARS-CoV-2 infection (confirmed by anti-N Ig) excluded	Anti-RBD Ig (chemiluminescence microparticle antibody assay) Neutralizing antibody activity (microneutralization assay) SARS-CoV-2–specific T-cell response (quantification by ELISA of IFN-γ and IL-2 production in response to stimulation of whole blood with a pool of overlapping SARS-CoV-2 spike protein peptides)	Similar humoral and T-cell responses in PLWH with good CD4 + T-cell counts as compared to HIV-negative controls Both humoral and T-cell responses significantly poorer in PLWH with current CD4 + T-cell counts < 200/μL
Woldemeskel et al*.* [97]	BNT162b2 (2 doses)	Observational study (USA)	12 PLWH: 5 males, 7 females; median age: 52 years (IQR: 25–59) 17 HIV-negative controls: 10 males, 7 females; median age: 41 years (IQR: 24–59)	Median CD4 + T-cell count: 913/μL (IQR: 649–1678) Plasma HIV-RNA > 20 copies/mL in 3 individuals despite cART	No evidence of prior SARS-CoV-2 infection by history or serology	Anti-S1 IgG (ELISA) ACE2 blocking activity (spike-ACE2 binding inhibition assay) SARS-CoV-2–specific T-cell response (IFN-γ ELISpot after stimulation of PBMCs with a pool of overlapping SARS-CoV-2 spike protein peptides)	Comparable humoral and T-cell responses in PLWH and HIV-negative controls Higher antibody titers in women compared to men
Tuan et al*.* [124]	BNT162b2 (2 doses)	Observation cohort study (USA)	26 PLWH: 16 (62%) males, 10 (38%) females; median age: 61 years	CD4 T-cell count > 200/μL: 24 (92%) Plasma HIV-RNA: - undetectable: *n* = 20 - detectable < 100 copies/mL: *n* = 6	Individuals with prior laboratory-confirmed or breakthrough COVID-19 excluded	Anti-RBD IgG (ELISA) SARS‑CoV‑2–specific T-cells (intracellular cytokine staining (ICS) assay and activation induced marker (AIM) assay by flow cytometry after stimulation of PBMCs with a pool of overlapping SARS-CoV-2 spike protein peptides) Time-points: 3 weeks post first vaccination; 2 weeks post second vaccination; 6 months post-first vaccination	Anti-RBD IgG detectable in 100% PLWH with a median 3.305-fold decrease 6 months after vaccination SARS-CoV-2–specific T-cell responses also present 6 months after primary vaccination
Sisteré-Oró et al*.* [98•]	BNT162b2 (2 doses)	Observational study (Spain)	10 immunological non-responder (INR) PLWH: 7 (70%) males; median age: 49 years (IQR: 30–71) 10 HIV-negative controls: 4 (40%) males; median age: 47 years (IQR: 26–72)	CD4 + T-cell count strata: - < 200/μL: *n* = 8 (80%) - 200–349: *n* = 2 (20%) All PLWH on ART for ≥ 6 months Undetectable plasma HIV-RNA: *n* = 8 (80%)	No evidence of prior SARS-CoV-2 infection	Anti-S IgG (ELISA) Neutralizing antibody activity (NeutraLISA assay) SARS-CoV-2–specific T-cells (ELISpot after stimulation of PBMCs with a pool of overlapping SARS-CoV-2 spike protein peptides) Time-points: pre-vaccination, post-vaccination, and 3 weeks after an additional boost in 3 previous non-responders	Insufficient immune responses in 50% of INR-PLWH compared to the HIV-negative controls Vaccine responsiveness not directly linked to CD4 + T cell counts Three of the five non-responders who agreed to receive a booster vaccination subsequently generated a SARS-CoV-2–specific T-cell response
Levy et al*.* [88]	BNT162b2 (2 doses)	Prospective open study (Israel)	143 PLWH: 131 males, 12 females; median age: 49.8 years 261 HIV-negative controls: 66 males, 195 females; median age: 55.8 years	Median CD4 + T-cell count at baseline: 700/μL (95% CI 648–757) All PLWH on cART Undetectable plasma HIV-RNA: 95%	Not part of study criteria	Anti-RBD IgG (ELISA) Neutralizing antibody activity (pseudovirus neutralization assay)	Comparable humoral responses in PLWH and HIV-negative controls
Jedicke et al*.* [125]	BNT162b2 (2 doses)	Observational study (Germany)	88 PLWH who received at least one dose: 86 males, 14 females; median age: 53.5 years (IQR: 26–86) 52 PLWH who received two doses: 73 males, 27 females; median age: 60.2 years (IQR: 32–85) 41 HIV-negative controls	Median CD4 + T cell count in PLWH who received at least one dose: 716/μL (151–1558) CD4 + T cell count in PLWH who received two doses: 577/μL (45–1106) Plasma HIV-RNA: - < 200 copies/mL: 100% - < 50 copies/mL: 96.5%	No previous infection with SARS-CoV-2	Anti-S IgG (ELISA) Neutralizing antibody activity (surrogate virus neutralization test)	Humoral responses after the first dose lower in PLWH with a CD4/CD8 ratio < 0.5 Robust humoral immunity in the majority of PLWH irrespective of CD4 + T-cell nadir, current CD4 + T-cell count or CD4/CD8 ratio after the second dose Significantly higher mean anti-S IgG concentrations with less variability in HIV-negative controls
Milano et al*.* [126]	BNT162b2 (2 doses)	Retrospective observational study (Italy)	697 PLWH: 521 (74.7%) males; median age: 53 years (IQR: 19 − 79)	Median CD4 + T-cells percentage: 35% (IQR: 0 − 59.2) CD4 + T-cell count < 200/μL: *n* = 14 (2.0) 696 (99.8%) participants on cART Undetectable plasma HIV‐RNA: *n* = 632 (90.7%)	PLWH recovered from SARS‐CoV‐2 infection within 3 months or with active infection at the time of vaccination (as shown by positive PCR on respiratory swabs or for history) excluded	Anti‐RBD IgG (chemiluminescent microparticle immunoassay) Time-points: T0 = first dose; T2 = second dose; T3 = 1 month after the second dose; T4 = 3 months after the second dose	Positive anti-RBD IgG titers in 94.1% at T2, 99.8% at T3, and 98.6% at T4 No correlation between humoral responses to vaccine and viro-immunological characteristics (including CD4 + T-cell counts)
Tau et al*.* [99]	BNT162b2 (2 doses)	Prospective study (Israel)	136 PLWH: 109 (79.6%) males, 28 (20.4%) females; median age: 44 years (IQR: 37–52) 61 HIV-negative controls: 52% males; median age: 49 years (IQR: 42–63)	Mean CD4 + T-cell count: 756/μL (SD: 314) CD4 + T-cell count < 300/μL: *n* = 9 Plasma HIV-RNA undetectable for most of the participants (95.6%)	No evidence of previous infection with SARS-CoV-2	Anti-RBD IgG (chemiluminescent microparticle immunoassay) SARS‑CoV‑2–specific T-cells (intracellular cytokine staining (ICS) by flow cytometry after stimulation of PBMCs with a pool of overlapping SARS-CoV-2 spike protein peptides)	Comparable humoral and T-cell responses in PLWH and HIV-negative controls Lower antibody titers in PLWH with CD4 + T-cell counts < 300/μL
Schmidt et al*.* [127]	BNT162b2 (2 doses)	Prospective observational study (Germany)	50 vaccinated PLWH: 34 males, 16 females; median age: 55 years (IQR: 46–60) 60 vaccinated HIV-negative controls: 32 males, 28 females; median age: 42 years (IQR: 30–53) 26 convalescent PLWH 17 convalescent HIV-negative controls	Median CD4 + T-cell count in vaccinated PLWH: 634/μL (IQR: 370–906) CD4 + T-cell count < 300/μL in vaccinated PLWH: *n* = 5 Median CD4 + T-cell count in convalescent PLWH: 551/μL (IQR: 382–761) CD4 + T-cell count < 300/μL in convalescent PLWH: *n* = 3 Plasma HIV-RNA < 20 copies/mL in all participants	26 PLWH and 17 HIV-negative controls with previous COVID-19 enrolled	Anti-S IgG/IgA (ELISA) Neutralizing antibody activity (NeutraLISA)	Lower neutralizing capacity and anti-S IgA in PLWH Levels of anti-S IgG and neutralizing activity higher in vaccinees compared to convalescents, irrespective of HIV status
Spinelli et al*.* [100]	BNT162b2 or mRNA-1273 (2 doses)	Case–control observational study (USA)	100 PLWH: 78 males, 13 females; median age: 59 years (IQR: 50–66) 100 HIV-negative controls: 78 males, 13 females; median age: 59 years (IQR: 52–66)	Median CD4 + T-cell count: 511/μL (IQR: 351–796) Plasma HIV-RNA > 200 copies/mL: *n* = 5	Individuals with prior SARS-CoV-2 infection excluded	Anti-RBD IgG (ELISA) Neutralizing antibody activity (surrogate virus neutralization assay)	Lower surrogate virus neutralization test response and trend towards lower anti-RBD IgG in PLWH, particularly among those with lower CD4 + T-cell counts and who received the BNT162b2 vaccine
González de Aledo et al*.* [128]	mRNA-1273 or BNT162b2 (2 doses)	Observational study (Spain)	100 PLWH: 75 (75%) males; age: 44 ± 10 years	CD4 + T-cell count: 785 ± 407/μL CD4 + T-cell count > 200/μL: *n* = 96 (96%) Plasma HIV-RNA < 50 copies/mL: *n* = 98 (98%)	No evidence of SARS-CoV-2 infection	Anti-S (trimeric) IgG (ELISA)	Seroconversion in all participants; low antibody concentration (< 520 BAU/mL) only in 3 vaccinees
Vergori et al*.* [102••]	BNT162b2 or mRNA-1273 (3^rd^ dose)	Observational cohort study (Italy)	216 PLWH with advanced disease (CD4 + T-cell count < 200/μL and/or previous AIDS): 39 (18.1%) females; median age: 54 years (IQR: 47–59) 98 HIV-negative controls: 72 (73.5%) females; median age: 44 years (IQR: 32–52)	CD4 + T-cell count strata: - > 500/μL (High CD4 + T-cell recovery): *n* = 76 - 201–500/μL (Intermediate CD4 + T-cell recovery): *n* = 96 - < 200/μL (Poor CD4 + T-cell recovery): *n* = 44 HIV-RNA < 50 copies/mL: 92.6%	Individuals with a SARS-CoV-2 infection diagnosis excluded	Anti-RBD IgG (chemiluminescence microparticle antibody assay) Neutralizing antibody activity (micro-neutralization assay) SARS-CoV-2–specific T-cell response (quantification by ELISA of IFN-γ production in response to stimulation of whole blood with a pool of overlapping SARS-CoV-2 spike protein peptides)	After the third dose, strong increase of humoral but not T-cell responses (as compared to values achieved after the second dose) in PLWH presenting with advanced disease at the time of HIV diagnosis, irrespective of the current CD4 + T-cell count Higher responses in PLWH who received mRNA-1273 as third dose following two doses of BNT162b2
Vergori et al*.* [129•]	BNT162b2 or mRNA-1273 (3^rd^ dose)	Observational cohort study (Italy)	106 PLWH, divided into two groups: low CD4 + *nadir* group (< 350/μL) and high CD4 + *nadir* group (> 350/μL) PLWH in the CD4 + low *nadir* group described according to CD4 + T-cell count strata: < 200/μL: 8.6% females; median age: 60 years (IQR: 52–68) 201–500/μL: 4.9% females; median age: 56 years (IQR: 47–61) > 500/μL: 4.9% females; median age: 56 years (IQR: 48–60) 28 HIV-negative controls: 8.9% females; median age: 46.5 years (IQR: 34–53)	CD4 + T-cell count strata in the CD4 + low *nadir* group: - < 200/μL: *n* = 27 (33%) - 201–500/μL: *n* = 29 (36%) - > 500/μL: *n* = 25 (31%)	Individuals with prior SARS-CoV-2 infection excluded	Neutralizing antibody titers (microneutralization assay)	After the third dose, strong increase in the neutralizing activity against Omicron variant (BA.1) – which remains poorer than that against the original W-D614G strain –, regardless of HIV status
Ruddy et al*.* [130]	BNT162b2 or mRNA-1273 (2 doses)	Prospective observational cohort study (USA)	14 PLWH: 13 (93%) males; median age: 62 years (IQR: 56–70)	CD4 + T-cell counts strata: - < 200: *n* = 2 - 200–349: *n* = 1 - 350–499: *n* = 3 - > 500: *n* = 8 All PLWH on cART for at least 6 months Undetectable plasma HIV-RNA: *n* = 13 (93%)	No pre-vaccination history of COVID-19	Anti-RBD IgG/IgM (ELISA)	High binding antibody titers in PLWH with well-controlled HIV on cART, regardless of CD4 + T-cell counts
Chammartin et al*.* [118]	BNT162b or mRNA-1273 (2 doses)	Observational cohort study (Switzerland)	333 PLWH: 65 (20.2%) females; age > 60 years: *n* = 231 (72%)	CD4 + T-cell counts > 350/μL: *n* = 298 (92.8%) HIV-RNA < 50 copies/mL: *n* = 13 (4%)	Individuals with prior SARS-CoV-2 infection excluded	Antibody response to SARS-CoV-2 [Antibody Coronavirus Assay 2 (ABCORA 2), which also allows for a reliable prediction of neutralization activity against the SARS-CoV-2 Wuhan-Hu-1 strain based on anti-S1 reactivity]	Antibody response higher among PLWH < 60 years, with CD4 + cell count > 350 cells/mL and vaccinated with mRNA-1273 compared with BNT162b2 Pre-infection with SARS-CoV-2 boosted the antibody response
Benet et al*.* [101•]	BNT162b2 or mRNA-1273 (2 doses)	Prospective observational single-center cohort study (Spain)	94 PLWH, described according to CD4 + T-cell count strata: < 200/μL: 79.3% males; median age: 52 years (IQR: 40–56) > 500/μL: 80.6% males; median age: 51 years (IQR: 40–56) 33 HIV-negative controls: 18 (54.5%) males; median age: 53 years (IQR: 35–57)	CD4 + T-cell strata: - < 200/μL: *n* = 58 - > 500/μL: *n* = 36 Undetectable plasma HIV-RNA: - CD4 + < 200/μL group: *n* = 47 (81%) - CD4 + > 500/μL group: *n* = 35 (100%)	Individuals with known history of SARS-CoV-2 infection excluded	Anti-S IgG and anti-RBD IgG (ELISA) Neutralizing antibody activity (neutralization assay) SARS-CoV-2 specific T-cells (ELISpot after stimulation of PBMCs with a pool of overlapping SARS-CoV-2 peptides)	Lower anti-S and anti-RBD IgG levels in PLWH with CD4 + T-cell counts < 200/μL Neutralizing capacity and specific T-cell responses (against wild-type SARS-CoV-2 and VOCs like Alpha, Delta, Kappa) absent or reduced in a higher percentage of PLWH with CD4 + T-cell counts < 200/μL
Woldemeskel et al*.* [123•]	PLWH: BNT162b2 (2 doses) HIV-negative controls: mRNA-1273 (2 doses)	Prospective observational study (USA)	8 PLWH: age range: 41–60 years 25 HIV-negative controls: age range: 21–60 years	Median CD4 + T-cell count: 1044/μL (IQR: 468–1420) All PLWH on cART 6 PLWH had plasma HIV-RNA < 20 copies/mL, while 2 PPLWH had low level viremia (49 and 52 copies/mL, respectively)	Not part of study criteria	Anti-S1 IgG (ELISA) ACE2-inhibiting antibodies (spike-ACE2 binding inhibition assay) SARS-CoV-2 specific T-cells (ELISpot after stimulation of PBMCs with a pool of overlapping SARS-CoV-2 spike protein peptides) Time-points: 2 weeks and 6 months after the second dose of vaccination	Similar to HIV-negative controls, waning antibody responses (especially to VOCs) but persistent T-cell responses 6 months post vaccination in PLWH
Frater et al*.* [105••]	ChAdOx1-S (2 doses)	Single-arm substudy of a phase 2/3 clinical trial (UK)	54 PLWH: 54 (100%) males; median age: 42.5 years (IQR: 37.5–49.8) 50 HIV-negative controls: 26 (52%) males, 24 (48%) females; median age: 38.5 years (IQR: 29.2–45.0)	Median CD4 + T-cell count: 694/μL (IQR: 574–860) All PLWH on cART for at least 3 months Plasma HIV-RNA < 50 copies/mL in all participants	Participants with previous SARS-CoV-2 infection (confirmed by anti-N IgG) excluded	Anti-S (trimeric) IgG (ELISA) Neutralizing antibody activity (focus reduction neutralization assay) SARS-CoV-2–specific T-cells (ELISpot after stimulation of PBMCs with a pool of overlapping SARS-CoV-2 spike protein peptides; T-cell proliferation assay by flow cytometry after stimulation of PBMCs with a pool of overlapping SARS-CoV-2 spike protein peptides) Time-points: 14, 28, 42, and 56 days after prime vaccination	Comparable humoral and T-cell responses (in terms of magnitude and persistence) in PLWH and HIV-negative controls No correlation between magnitude of anti-S IgG and CD4 + T-cell counts or age
Ogbe et al*.* [106]	ChAdOx1-S (2 doses)	Open-label non-randomized group within the phase II/III COV002 trial (UK)	54 PLWH: 54 (100) male, median age 42.5 years (IQR 37.5–49.8) 50 HIV negative (ELISA and ELISpot assay): 26 (52%) males, 24 (48%) females, median age 38.5 years (IQR 29.2–45.0) 10 HIV negative (ELISA and ELISpot assay): 10 (100%) males,	Median CD4 + T-cell count: 694/μL (IQR: 574–860) Plasma HIV-RNA < 50 copies/mL in all participants	No evidence of previous infection with SARS-CoV-2	Anti-S (trimeric) IgG (ELISA) IgG responses to SARS-CoV-2, SARS-CoV-1, MERS-CoV, and seasonal coronaviruses (MSD binding assay) Neutralising antibody activity (focus reduction neutralization assay; ACE2 inhibition assay) SARS-CoV-2–specific T-cells (ELISpot after stimulation of PBMCs with a pool of overlapping SARS-CoV-2 spike protein peptides; T-cell proliferation assay and AIM assay by flow cytometry after stimulation of PBMCs with a pool of overlapping SARS-CoV-2 spike protein peptides) Time-points: 0, 14, 28, 42, 56, and 182 days after prime vaccination	Similar humoral and T-cell responses in PLWH and HIV-negative controls Decline of both humoral and T-cell responses 6 months post vaccination in both PLWH and HIV-negative controls Immune responses to VOCs detectable but lower than those to wild-type Preexisting cross-reactive T-cell responses to SARS-CoV-2 spike (correlated with prior exposure to beta coronaviruses) associated with greater postvaccine immunity
Madhi et al*.* [107]	ChAdOx1-S (2 doses)	Multicenter, randomized, double-blind, placebo-controlled, phase 1B/2A COV005 trial (South Africa)	102 PLWH: 26% males, 74% females; median age: 40 years (IQR: 33–46); 52 received vaccine and 50 placebo 56 HIV-negative controls: 62% males, 38% females; median age: 32 years (IQR: 25–42); 28 received vaccine and 28 placebo	Median CD4 + T-cell count: 695/μL (IQR: 512–929) All PLWH on cART for at least 3 months HIV-RNA < 50 copies/mL: *n* = 27 (75%)	Baseline SARS-CoV-2 seropositive and seronegative participants included	Anti-RBD and anti-S IgG (ELISA) Neutralising antibody activity (pseudovirus neutralization assay) Time-points: 0, 28, and 42 days after prime vaccination	Comparable IgG (against D614G wild type and Beta variant) in PLWH and HIV-negative controls Higher antibody responses after each vaccine dose in PLWH with previous SARS-CoV-2 infection as compared to SARS-CoV-2–naïve PLWH PLWH who develop high-titre IgG responses also retain neutralization activity against Beta
Khan et al*.* [66]	Ad26.CoV2.S (1 dose)	Prospective cohort study (South Africa)	26 PLWH: - 34 SARS-CoV-2–experienced unvaccinated - 18 SARS-CoV-2–experienced vaccinated - 8 SARS-CoV-2–naïve vaccinated 73 HIV-negative controls: - 28 SARS-CoV-2–experienced unvaccinated - 49 SARS-CoV-2–experienced vaccinated - 24 SARS-CoV-2–naïve vaccinated	All PLWH receiving cART SARS-CoV-2–experienced unvaccinated: - median CD4 + T-cell count: 581/μL (IQR: 328–794) - detectable plasma HIV-RNA in 10 individuals SARS-CoV-2–experienced vaccinated: - median CD4 + T-cell count: 1033/μL (IWR: 877–1424) - detectable plasma HIV-RNA in 1 individual SARS-CoV-2–naïve vaccinated: - 735/μL (IQR: 458–863) - undetectable plasma HIV-RNA in all individuals	Individuals with prior SARS-CoV-2 infection included	Delta variant neutralization capacity (live virus neutralization assay)	Delta variant neutralization capacity in vaccinated PLWH with well-controlled HIV infection not inferior to HIV-negative controls, irrespective of past SARS-CoV-2 infection Reduced Delta variant neutralization capacity in unvaccinated PLWH with prior SARS-Cov-2 infection (particularly in those with uncontrolled HIV viremia) compared to HIV-negative controls
Noe et al*.* [114]	BNT162b2 (2 doses) or mRNA-1273 (2 doses) or ChAdOx1-S (2 doses) or Ad26.CoV2.S (1 dose) or heterologous vaccination (1 dose of ChAdOx1-S + 1 dose of mRNA vaccine)	Observational retrospective study (Germany)	590 PLWH vaccinated with mRNA vaccine (BNT162b2 or mRNA-1273): 492 males, 8 females; median age: 52 years (IQR: 43–59) 31 PLWH vaccinated with ChAdOx1-S: all males; median age: 31 years (IQR: 49.5–63) 15 PLWH vaccinated with Ad26.CoV2.S: 12 males, 3 females; median age: 46 years (IQR: 39.5–59) 29 PLWH vaccinated with heterologous schedule: 25 males, 4 females; median age: 56 years (IQR: 48–59)	Median CD4 + T-cell count: 708/μL (IQR: 524–912) Median *nadir* CD4 + T-cell count: 264/μL (IQR: 143.5–88.2) Plasma HIV-RNA < 50 copies/mL: *n* = 622 (93.5%)	Participants with prior SARS-CoV-2 infection excluded	Anti-S (trimeric) IgG (ELISA)	Robust antibody responses in PLWH Factors associated with higher humoral responses to vaccines: high CD4 + T-cell counts, mRNA vaccination scheme, being female
Hassold et al*.* [131]	BNT162b2 (2 doses) or mRNA-1273 (2 doses) or ChAdOx1-S (2 doses)	Observational retrospective study (France)	105 PLWH: 35.2% females; median age: 54 years (IQR: 46–60), described according to CD4 + T-cell count strata: - < 200/μL: 9 (50%) males; median age: 52.5 years (IQR:39.3–57.5) - 200–500/μL: 24 (66%) males; median age: 54.9 years (IQR: 46.9–59.6) - > 500/μL: 35 (68%) males; median age: 54.2 years (IQR: 47.1–61.4)	CD4 + T-cell strata: - < 200/μL: *n* = 18 - 200–500/μL: *n* = 36 - > 500/μL: n = 51 Plasma HIV-RNA < 50 copies/mL: - CD4 + < 200/μL group: *n* = 8 (44.4%) - CD4 + = 200–500/μL group: *n* = 30 (83.3%) - CD4 + > 500/μL group: n = 49 (96%)	Participants with prior SARS-CoV-2 infection excluded	Anti-RBD IgG (chemiluminescent microparticle immunoassay)	Lower humoral responses in PLWH with CD4 + T-cell counts < 500/μL, and even lower in those with CD4 + T-cell counts < 200/μL
Lapointe et al*.* [115]	Initial 2-dose regimen: - mRNA + mRNA - ChAdOx1-S + mRNA - ChAdOx1-S + ChAdOx1-S 3rd dose: - BNT162b2 - mRNA-1273	Observational retrospective study (Canada)	99 PLWH: 87 (88%) males; median age: 54 years (IQR: 40–61) 152 HIV-negative controls: 50 (33%) males; median age: 47 years (IQR: 35–70)	Median CD4 + T-cell count: 715/μL (IQR: 545–943) Median *nadir* CD4 + T-cell count: 280/μL (IQR: 123–490) All PLWH on cART Plasma HIV-RNA < 50 copies/mL in all participants	No previous infection with SARS-CoV-2	Anti-RBD IgG (ELISA) Neutralizing antibody activity (live virus neutralization assay; ACE2 receptor displacement assay) Time-points: before vaccination; 1 month after the first dose; 1, 3, and 6 months after the second dose; 1 month after the third dose	No evidence of lower antibody concentrations or faster rates of antibody decline in PLWH compared with HIV-negative controls No evidence of poorer viral neutralization in PLWH after 2 doses No correlation between humoral responses to vaccine and CD4 + T-cell *nadir* Post–third-dose humoral responses comparable or higher in PLWH compared to HIV-negative controls Omicron-specific responses weaker than responses against wild-type virus Higher humoral responses post third dose in PLWH who received mRNA-1273
Corma-Gómez et al*.* [116•]	BNT162b (2 doses) or mRNA-1273 (2 doses) or ChAdOx1-S (2 doses) or Ad26.COV2.S (1 dose)	Multicenter prospective cohort study (Spain)	420 PLWH: 343 (82%) males; median age: 55 years (IQR: 49–60)	Median CD4 + T-cell count: 586/μL (IQR: 380–786) CD4 + T-cell count strata: - > 350/μL: *n* = 326 - 200–349/μL: *n* = 61 - < 200/μL: *n* = 33 Plasma HIV-RNA < 50 copies/mL: *n* = 362 (87%)	Patients with documented prior SARS-CoV-2 natural infection (diagnosed by PCR, antigen detection, or serology) excluded	Anti-S1/S2 IgG (chemiluminescent immunoassay) Neutralizing antibody activity (indirect chemiluminescent immunoassay) Time-points: 4–8 weeks after the last dose	Lower humoral responses in PLWH with low CD4 + T-cell counts mRNA vaccines associated with a higher response than adenoviral vector vaccines
Brumme et al*.* [117]	2-dose regimen: - mRNA + mRNA - ChAdOx1-S + mRNA - ChAdOx1-S + ChAdOx1-S	Longitudinal observational study (Canada)	100 PLWH: 88 (88%) males; median age: 54 years (IQR: 40–61) 152 HIV-negative controls: 50 (33%) males; median age: 47 years (IQR: 35–70)	CD4 + T-cell count: 710/μL (IQR: 525–935) Plasma HIV-RNA < 50 copies/mL in all participants	8% of PLWH and 10% of HIV-negative controls with prior SARS-CoV-2 infection identified at baseline screening (by anti-N antibodies)	Anti-RBD IgM/IgG (electro-chemiluminescence assay) Neutralising antibody activity (live virus neutralization assay; ACE2 displacement assay) Time-points: prior to vaccination; 1 month after the first dose; 1 and 3 months after the second dose	Lower anti-RBD antibodies and ACE2 displacement activity after the first dose in PLWH compared to HIV-negative controls Comparable humoral responses after the second dose in the two groups No correlation between humoral responses to vaccine in PLWH and CD4 + T-cell current count or *nadir* Factors associated with lower humoral responses to vaccination in PLWH: older age, higher burden of chronic health conditions, and dual ChAdOx1-S vaccination
Madhi, et al*.* [108•]	NVX-CoV2373 (2 doses)	Randomized, observer-blinded, placebo-controlled, phase 2A/B trial (South Africa)	244 PLWH: - vaccine group: *n* = 122; 37 (30.3%) males, 85 (69.7%) females; median age: 38 years (IQR: 20–60) - placebo group: *n* = 122; median age: 38 years (IQR: 20–59); 29 (23.8%) males, 93 (76.2%) females 4164 HIV-negative controls: - vaccine group: *n* = 2089; median age: 27 years (IQR: 18–84); 1217 (58.3%) males, 872 (41.7%) females - placebo group: *n* = 2075; median age: 27 years (IQR: 18–83); 1239 (59.7%) males, 836 (40.3%) females	CD4 + T-cell count: - vaccine group: 729.5/µL (IQR: 80–2076) - placebo group: 744/µL (IQR: 182–1799) Plasma HIV-RNA: - vaccine group: 68.5 copies/mL (IQR: 0–735) - placebo group: 62 copies/mL (IQR: 20–628) PLWH on cART for at least 8 weeks	PLWH: - vaccine group: 43 (35.2%) experienced; 79 (64.8%) naïve - placebo group: 40 (32.8%) experienced; 82 (67.2%) naïve HIV-negative controls: - vaccine group: 692 (33.1%) experienced; 1397 (66.9%) naïve - placebo group: 730 (35.2%) experienced; 1345 (64.8%) naïve	Anti-S IgG (ELISA) Neutralizing antibody activity (live virus neutralization assay; spike-ACE2 binding inhibition assay) Time-points: before vaccination; 21 days after first dose; 14 days after second dose Immunological analyses on: 101/2046 PLWH receiving the vaccine and 103/2044 PLWH receiving the placebo; 1945/3886 controls receiving the vaccine and 1941/3886 receiving the placebo	SARS-CoV-2–naïve: humoral responses lower in PLWH as compared to HIV-negative controls SARS-CoV-2–experienced: before vaccination similar levels of humoral responses between PLWH and HIV-negative controls, which remained similar across time points
Liu, et al*.* [109]	CoronaVac (2 doses)	Cross-sectional, observational study (China)	55 PLWH: all males; mean age: 36 years (SD: 11) 21 HIV-negative controls: 17 (81%) males, 4 (19%) females; mean age: 35 years (SD: 8)	CD4 + T-cell count strata: - ≥ 350/µL: immunological responders - < 350/µL: immunological non-responders All PLWH on cART for at least 1 year HIV-RNA < 50 copies/mL in all participants	Participants with prior SARS-CoV-2 infection excluded	Anti-RBD IgG (ELISA) Time-points: 2–18 weeks after the second dose	Similar anti-RBD IgG in PLWH and HIV-controls Lower humoral response in PLWH with CD4 + T-cell counts < 350/µL
Han, et al*.* [111]	CoronaVac or BBIBP-CorV (2 doses)	Non-interventional study (China)	47 PLWH: 45 (95.7%) males, 4 (4.3%) females; median age: 34 years (IQR: 26–42) 18 HIV-negative controls: all males; median age: 40 years (IQR: 28–57)	Median CD4 + T-cell count: 474 cells/µL (IQR: 145–926) CD4 + T-cell count strata: - ≤ 350 cells/µL: suboptimal immune recovery - > 350 cells/µL: good immune recovery All PLWH on cART for at least 6 months Median plasm HIV-RNA: 20 copies/mL (IQR: 20–163.8)	Participants with prior SARS-CoV-2 infection excluded	Anti-S IgG (magnetic particles chemiluminescence immunoassay) Neutralizing antibody activity (pseudovirus neutralization assay) Time–points: 28 days after vaccination	Lower levels of IgG and neutralization activity (against both D614G and Delta variant) in PLWH compared to HIV-negative controls Even lower humoral responses in PLWH with low CD4 + T-cell counts
Feng, et al*.* [110•]	BBIBP-CorV (2 doses)	Open-label two-arm non-randomized study (China)	42 PLWH: 29 males, 13 females; mean age: 42.7 years 28 HIV-negative controls: 16 males, 12 females; mean age: 37.8 years	Mean CD4 + T-cell count: 659/µL (SD: 222) All PLWH on cART Plasma HIV-RNA: - > 20 copies/mL: *n* = 12 - < 20 copies/mL: *n* = 8 - undetectable: *n* = 22	Participants with prior SARS-CoV-2 infection excluded	Anti-RBD Ig (ELISA) Neutralizing antibody activity (chemiluminescent reaction; spike-ACE2 binding inhibition assay) SARS-CoV-2–specific T-cells (ICS by flow cytometry after stimulation of PBMCs with spike peptide pool) Time-points: baseline; 4 weeks after first dose; 4 weeks after second dose	Similar anti-RBD Ig, neutralization activity and T-cell responses between PLWH and HIV-negative controls Lower humoral responses in PLWH with low CD4/CD8 ratio compared to PLWH with medium or high ratio
Cai, et al*.* [132]	BBIBP-CorV or CoronaVac (2 doses)	Single-centre cross-sectional study (China)	143 PLWH: 140 (97.9%) males, 3 (2.1%) females; mean age 32.55 years (SD: 8.69) 50 HIV-negative controls: 48 (96%) males, 2 (4%) females; mean age: 29.84 (SD: 8.51)	Mean CD4 + T-cell count: 398.96/µL (SD: 202.31) 116 (81.1%) PLWH on cART	Individuals with prior SARS-CoV-2 infection included	Anti-RDB IgG, anti-N IgM (magnetic chemiluminescence enzyme assay) Neutralizing antibody activity (live virus neutralization assay) Time-points: 64.46 ± 41.22 or 35.78 ± 27.99 days after vaccination in HIV-negative controls and PLWH, respectively	Anti-RBD IgG levels lower in PLWH than HIV-negative controls Positive correlation between anti-RBD IgG and CD4 + T-cell counts Anti-N IgM levels and neutralization ability against WT and Delta variant similar between groups
Zeng, et al*.* [112]	BBIBP-CorV or CoronaVac (2 doses)	Longitudinal study (China)	132 PLWH: 119 (90.2%) males, 13 (9.8%) females; mean age: 34.05 years (SD: 8.37) 130 HIV-negative controls: 115 (88.5%) males, 15 (11.5%) females; mean age: 34.4 years (SD: 8.9)	CD4 + T-cell count strata: - 200–349/µL: 14.4% - 350–499/µL: 26.5% - ≥ 500/µL: 49.2% cART: 87.9% of PLWH under stable treatment for more than 6 months; 7.6% less than 6 months; 4.5% without treatment Plasma HIV-RNA < 50 copies/mL: 90.9%	Participants with prior SARS-CoV-2 infection excluded	Anti-RBD IgG (magnetic particle luminescence assay) Time-points: 28 and 180 days after the second dose	Lower and less durable humoral responses in PLWH as compared to HIV-negative controls Positive correlation between humoral responses and CD4 + T-cell counts
Wong, et al*.* [113]	CoronaVac or BNT162b (2 doses)	Prospective longitudinal observational study (China)	PLWH: - CoronaVac: *n* = 423; 86% males, 14% females; median age: 50 years (IQR: 42–59) - BNT162b: *n* = 184; 93% males, 7% females; median age: 41 years (IQR: 34–50) HIV-negative controls: - CoronaVac: *n* = 32; 38% males, 63% females; median age: 38 years (IQR: 34–55) - BNT162b: *n* = 52; 58% males, 36.5 (32–43); 42% females; median age: 36.5 (IQR: 32–43)	Median CD4 + T-cell count:—- CoronaVac group: 566/µL (IQR: 382–745) - BNT162b group: 565/µL (IQR: 424–732) Plasma HIV-RNA < 30 copies/mL: - CoronaVac group: *n* = 130 (87%) - BNT162b group: *n* = 285 (87%)	Participants with prior SARS-CoV-2 infection excluded	Neutralizing antibody activity (surrogate virus neutralization test) Time-points: pre-second dose; 2 and 6 weeks post-second dose, 3 and 6 months post-second dose	Lower peak and shorter duration of sVNT responses following inactivated vaccine (CoronaVac) compared to mRNA vaccine in PLWH

## Concluding Remarks

COVID-19 pandemics posed a serious threat to public health and collided with HIV/AIDS, leading to a suboptimal care of PLWH worldwide. Nevertheless, concerns for higher susceptibility of PLWH to SARS-CoV-2 infection and poor COVID-19 outcomes arose, thus leading to prioritize this population for vaccination. Additionally, given the enduring immune dysfunction which features chronic HIV infection despite effective cART, PLWH have been considered at risk of lower and less functional immune responses to SARS-CoV-2 natural infection and vaccination, with potentially negative implications for disease outcomes and vaccines efficacy.

**Fig. 2:**
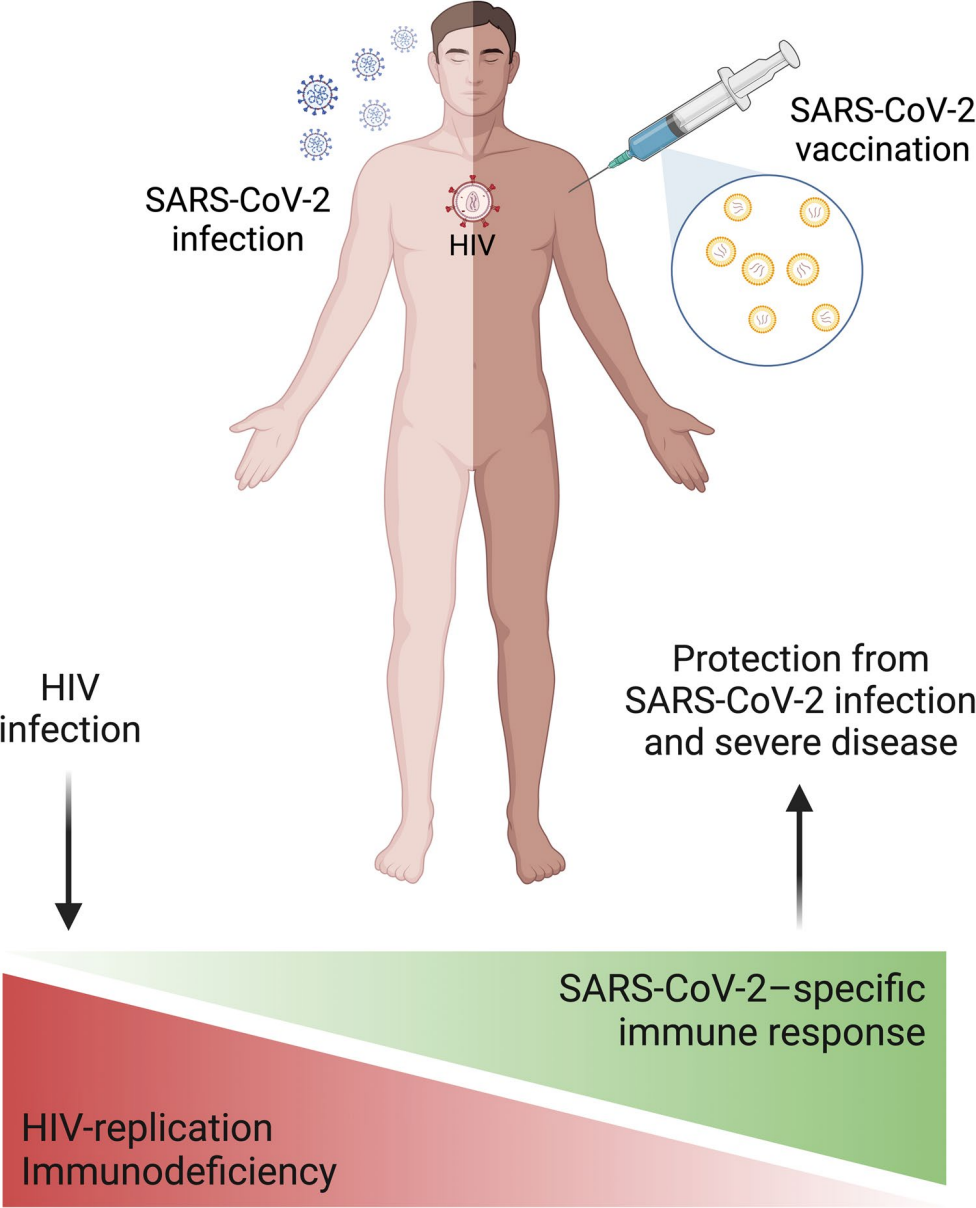
Interplay between HIV infection and immune responses to SARS-CoV-2 infection and vaccination. PLWH with well-controlled HIV infection on cART (suppressed HIV viremia and full recovery of CD4 + T-cell counts) mount adequate adaptive immune responses to both SARS-CoV-2 infection and vaccination, which lend protection against the severe forms of the disease and from future infections/reinfections. On the contrary, ongoing HIV replication and immunodeficiency with low CD4 + T-cell counts hamper the development of both T-cell and humoral memory in response to SARS-CoV-2 infection and vaccination, thus explaining the increased risk of severe COVID-19 and breakthrough infections in PLWH with scarce immunovirological control. Created with *BioRender.com*

Despite such concerns, current knowledge, albeit not granular and at times apparently conflicting, is somehow reassuring for PLWH with well-controlled HIV infection. Immune responses to both SARS-CoV-2 natural infection and vaccination in PLWH have been shown similar to those of people without HIV, with the only exception of those with low CD4 + T-cell counts and/or uncontrolled HIV viremia, who may indeed develop suboptimal T-cell and humoral immune memory following infection and vaccination (Fig. 2). Accordingly, COVID-19 severity and outcomes in this population seem to be worse especially in the presence of concurrent age-related comorbidities and in case of severe CD4 + T-cells depletion, a condition which also potentially increase the risk of breakthrough infections after the primary vaccine cycle, suggesting a suboptimal immune response to vaccination. Data regarding the risk of PASC among PLWH are extremely scarce, yet seemingly indicative of a higher incidence, albeit whether immune factors are involved in its pathogenesis and whether it can negatively impact the immunologic landscape of chronic HIV infection in the long run is yet to be determined.

Altogether, these data clearly support the need for additional vaccine doses in PLWH with ongoing HIV replication and/or scarce immune reconstitution despite virally effective cART. Furthermore, given the potentially higher risk of developing long-term sequelae, PLWH who experienced COVID-19 should be ensured a more careful and prolonged follow-up, in order to avoid adding the PASC fuel to the HIV crackling flames.
